# Tissue expression and developmental regulation of chicken cathelicidin antimicrobial peptides

**DOI:** 10.1186/2049-1891-3-15

**Published:** 2012-05-31

**Authors:** Mallika Achanta, Lakshmi T Sunkara, Gan Dai, Yugendar R Bommineni, Weiyu Jiang, Guolong Zhang

**Affiliations:** 1 Department of Animal Science, Oklahoma State University, Stillwater, OK, 74078, USA; 2 Current Address: Department of Microbiology, Xiangya School of Medicine, Central South University, Changsha, Hunan, 410078, China; 3 Current Address: Virology and Serology Section, Veterinary Diagnostic Services, New Mexico State Department of Agriculture, Albuquerque, NM, 87196, USA; 4 Department of Biochemistry and Molecular Biology, Oklahoma State University, Stillwater, OK, 74078, USA; 5 Department of Physiological Sciences, Center for Veterinary Health Sciences, Oklahoma State University, Stillwater, OK, 74078, USA

**Keywords:** Antimicrobial peptides, Cathelicidins, Chickens, Development, Host defense peptides

## Abstract

Cathelicidins are a major family of antimicrobial peptides present in vertebrate animals with potent microbicidal and immunomodulatory activities. Four cathelicidins, namely fowlicidins 1 to 3 and cathelicidin B1, have been identified in chickens. As a first step to understand their role in early innate host defense of chickens, we examined the tissue and developmental expression patterns of all four cathelicidins. Real-time PCR revealed an abundant expression of four cathelicidins throughout the gastrointestinal, respiratory, and urogenital tracts as well as in all primary and secondary immune organs of chickens. Fowlicidins 1 to 3 exhibited a similar tissue expression pattern with the highest expression in the bone marrow and lung, while cathelicidin B1 was synthesized most abundantly in the bursa of Fabricius. Additionally, a tissue-specific regulatory pattern was evident for all four cathelicidins during the first 28 days after hatching. The expression of fowlicidins 1 to 3 showed an age-dependent increase both in the cecal tonsil and lung, whereas all four cathelicidins were peaked in the bursa on day 4 after hatching, with a gradual decline by day 28. An abrupt augmentation in the expression of fowlicidins 1 to 3 was also observed in the cecum on day 28, while the highest expression of cathelicidin B1 was seen in both the lung and cecal tonsil on day 14. Collectively, the presence of cathelicidins in a broad range of tissues and their largely enhanced expression during development are suggestive of their potential important role in early host defense and disease resistance of chickens.

## Background

Antimicrobial peptides (AMPs) are an important component of the innate immune system playing a critical role in host defense and disease resistance in virtually all species of life [1-4]. AMPs generally consist of < 100 amino acid residues, mostly cationic and amphipathic in nature, which allows them to bind and disrupt negatively charged microbial membranes leading to cell death. Because of non-specific physical interactions with membranes, it is extremely difficult for bacteria to develop resistance. In addition to being antimicrobial, a number of AMPs were recently found to exert a broad range of immunomodulatory roles by recruiting and activating all major types of innate and adaptive immune cells [3,5]. Therefore, AMPs are being actively developed for the control and prevention of infectious diseases, particularly against antibiotic-resistant bacteria [3].

Cathelicidins are a major family of AMPs in vertebrate animals including chickens. All cathelicidins are synthesized as prepro-peptides, with the prepro-sequence being highly conserved across species and the carboxyl terminal, biologically active mature sequence highly diversified [6]. In mammals, besides the mucosal epithelial cells lining the digestive, respiratory, and reproductive tracts, cathelicidins are most abundantly expressed in myeloid progenitor cells and stored in neutrophil granules as pro-peptides, which are converted into active forms by proteolytic cleavage upon degranulation. The chicken genome was recently found to encode four genes for cathelicidins, namely fowlicidins 1 to 3 and cathelicidin B1 that span a 7.5-kb distance on chromosome 2 [7-10]. All four chicken cathelicidins are capable of killing a broad range of bacteria including antibiotic-resistant strains [9,11-14]. Like in mammals, fowlicidin-2/CMAP27 was shown to be localized in the granules of chicken heterophils, equivalent to mammalian neutrophils, and processed into a mature form upon stimulation with bacterial lipopolysaccharide [14].

AMPs including cathelicidins have been detected in the meconium and feces of human infants [15], and elevated levels of cathelicidin have been noticed in the infants associated with respiratory infections [16], suggestive of the role of cathelicidins and other AMPs in early host defense of humans. In chickens, expression of fowlicidins 1 to 3 is detected as early as day 3 of embryonic development and then significantly increased as the embryo develops further, whereas cathelicidin B1 is not expressed until day 9, but significantly increased by day 12 in the developing embryo [17].

In this study, we studied the tissue expression pattern of four cathelicidins in 28-day-old broiler chickens and further examined their expression in the first 28 days after hatching. We observed that chicken cathelicidin transcripts are synthesized in a wide range of tissues and differentially expressed during the development, suggesting that cathelicidins may play an important role of early host defense of chickens.

## Materials and methods

### Tissue sampling and preparation

Day-old male and female Cornish Rock broiler chickens were purchased from a commercial hatchery (Ideal Poultry, Cameron, TX, USA) and reared under standard care in Laboratory Animal Resource Facility at Oklahoma State University, Stillwater, OK, USA. Tissues were collected from chickens of 2, 4, 7, 14, and 28 days, with 3 to 5 animals per age group. The range of tissues that were harvested included the crop, esophagus, proventriculus, gizzard, duodenum, jejunum, ileum, cecal tonsil, cecum, colon, lung, heart, trachea, liver, spleen, thymus, kidney, skin, breast muscle, brain, testis, ovary and bursa. All tissues were snap frozen in liquid nitrogen and stored at −80°C until used. Animal procedures were approved by the Institutional Animal Care and Use Committee of Oklahoma State University under protocol no. AG0610.

### Isolation and quantification of total RNA

Tissues were homogenized in Tri Reagent (Sigma-Aldrich, St Louis, MO, USA), followed by total RNA extraction according to the manufacturer’s instructions. Air-dried RNA pellet was suspended in nuclease-free water and mixed thoroughly until the pellet was completely dissolved. RNA concentration and quality were measured using NanoDrop Spectrophotometer (NanoDrop Products, Wilmington, DE, USA).

### Reverse transcription of total RNA

QuantiTect Reverse Transcription Kit (Qiagen, Valencia, CA, USA) was used to synthesize the first-strand cDNA from total RNA following the manufacturer’s recommendations. Briefly, 0.3 μg of total RNA was first eliminated of genomic DNA contamination in a genomic DNA wipeout buffer for 5 min at 42°C. Reverse transcription was then performed in a total volume of 4 μL using Quantiscript reverse transcriptase and a mixture of random hexamers and oligo(dT) primers for 30 min at 42°C, followed by 3 min at 95°C to inactivate reverse transcriptase. The cDNA concentration was then measured using NanoDrop Spectrophotometer following a 10-fold dilution in nuclease-free water.

### Real time PCR

QuantiTect SYBR Green PCR Kit (Qiagen) was used for real-time amplification of the first-strand cDNA using MyiQ Real Time PCR Detection System (Bio-Rad, Hercules, CA, USA) as previously described [9]. Briefly, each PCR reaction was set up in a 96-well PCR plate in a total volume of 10 μL using 0.1 μg of the first-strand cDNA and gene-specific primers (Table 1). Real-time PCR was programmed as follows: initial denaturation at 95°C for 10 min, followed by 45 cycles of denaturation at 94°C for 15 s, annealing at 55°C for 20 s, and extension and data collection at 72°C for 30 s. Melting curve analysis was conducted to confirm the specificity of PCR amplifications. Comparative ∆∆C_t_ method was used for quantification of gene expression using the glyceraldehyde-3-phosphate dehydrogenase (GAPDH) gene as the reference gene for data normalization [9].

**Table 1:** Primer sequences of chicken cathelicidins for real time PCR

Gene	Forward primer (5' to 3')	Reverse primer (5' to 3')	Product size, bp	
			cDNA	Gene
Fowlicidin-1	GCTGTGGACTCCTACAACCAAC	GGAGTCCACGCAGGTGACATC	261	882
Fowlicidin-2	CAAGGAGAATGGGGTCATCAG	CGTGGCCCCATTTATTCATTCA	221	584
Fowlicidin-3	GCTGTGGACTCCTACAACCAAC	TGGCTTTGTAGAGGTTGATGC	352	1095
Cathelicidin B1	CCGTGTCCATAGAGCAGCAG	AGTGCTGGTGACGTTCAGATG	170	251
GAPDH	GCACGCCATCACTATCTTCC	CATCCACCGTCTTCTGTGTG	356	876

### Statistical analysis

All data were analyzed with one-way ANOVA, followed by Tukey’s test using GraphPad Prism 5 (GraphPad Software, La Jolla, CA, USA). The results were considered significant, if *P* < 0.05.

## Results and discussion

### Tissue expression pattern of chicken cathelicidins

**Figure 1:**
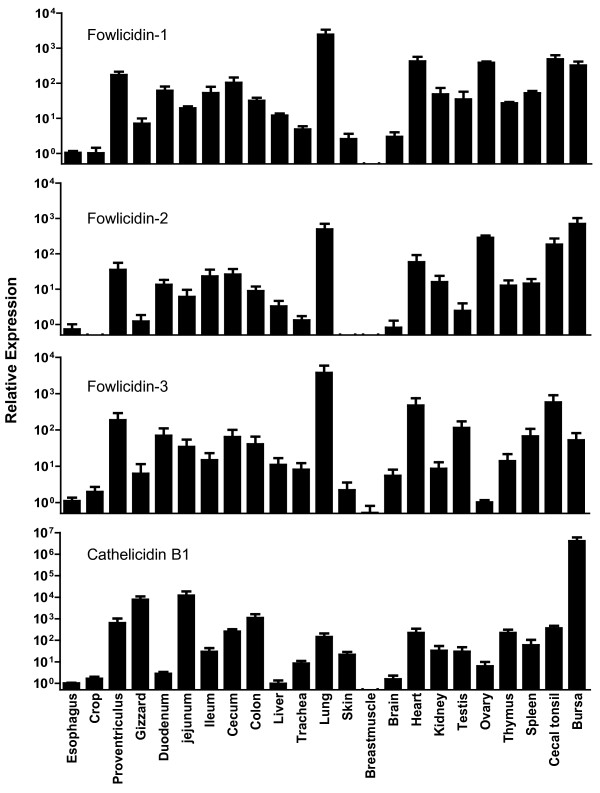
**Tissue Expression pattern of four chicken cathelicidins.** All tissues were collected from three 28-day-old broiler chickens and subjected to RNA isolation and real-time PCR using gene-specific primers. Expression levels of all tissues were calculated relative to that of the esophagus using GAPDH as a reference gene. Each bar represents mean ± standard error of three chickens.

To determine the expression pattern of chicken cathelicidins, a panel of tissues were collected from three 28-day-old Cornish Rock broiler chickens. Following RNA isolation and reverse transcription, real-time PCR was performed to reveal the gene expression levels of fowlicidins 1 to 3 and cathelicidin B1. As shown in Figure 1, all four cathelicidins were widely expressed in most tissues examined, except for breast muscle. It is evident that cathelicidin B1exhibited a distinct expression pattern from that of fowlicidins 1 to 3. While cathelicidin B1 was most abundantly expressed in the bursa of Fabricius, fowlicidins 1 to 3 were expressed highly in the lung and throughout the digestive tract (Figure 1). A similar expression profile of fowlicidins 1 to 3 is indicative of their close phylogenetic relationship, whereas cathelicidin B1 represents a distant family member. Indeed, fowlicidins 1 to 3 share a higher similarity at the amino acid sequence level than cathelicidin B1, although they reside in tandem in the same chromosomal region [9,10].

All four cathelicidin transcripts were found to be expressed in the testis and ovary (Figure 1). Additionally, heart showed an abundant expression of all four cathelicidins. It is noted that all primary and secondary lymphoid tissues including the bursa, thymus, spleen, and cecal tonsil express all four cathelicidins at moderate or high levels (Figure 1). In fact, bursa and bone marrow are the primary places for synthesis of cathelicidin B1 and fowlicidins 1 to 3, respectively (Figure 1) [10]. Cathelicidin B1 was found earlier to be expressed in secretory epithelial cells surrounding M cells, a major portal of entry for pathogens in mucosal lymphoid tissues including bursa [10]. It will be important to identify the cell types that express other cathelicidins in mucosal lymphoid tissues. At the same time, it will be interesting to reveal which cell type synthesizes cathelicidins in the spleen and thymus with no M cells present. Presence of cathelicidins in lymphoid tissues may be suggestive of a possible involvement of AMPs in the maturation and development of adaptive immunity. In fact, it is known that many AMPs including cathelicidins are capable of regulating adaptive immunity through activation of dendritic cells [18].

Expression of cathelicidins in a broad range of tissues including many non-immune tissue types also raised the possibility that these cathelicidins may play a role beyond host defense. Indeed, several AMPs have been shown to be involved in sperm maturation [19-21], consistent with the finding that a majority of defensins are most abundantly expressed in the male reproductive tracts in mammals [22].

As compared with mammalian species, chickens express a negligible amount of cathelicidins in the skin (Figure 1). This result could be attributed to the evolution of the skin in different species. The skin of birds is covered with feathers and many of the diseases in birds are contracted through oral and nasal routes. Hence, AMP synthesis in the skin might not be as much needed in birds than in mammals. In fact, a relatively high level of fowlicidin-2/CMAP27 expression was found in the uropygial gland [8], which secretes preen oil and antimicrobial factors that spread over plumage and provide protection against skin infections. On the other hand, as skin acts as an important route of entry for microorganisms in most mammals, it is not surprising to see a large amount of cathelicidins and other AMPs synthesized in the skin for protection.

### Developmental expression of chicken cathelicidins

**Figure 2:**
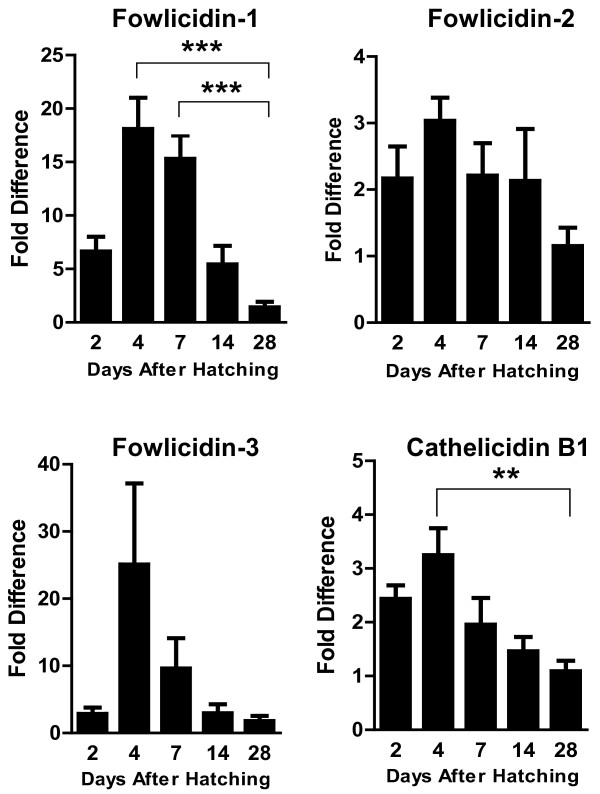
**Developmental regulation of chicken cathelicidins in the bursa of Fabricius.** Bursas were harvested from broiler chickens of indicated age and subjected to RNA isolation and real-time PCR analysis of the expression of four cathelicidins. Fold differences in the cathelicidin expression level among different days after hatching were calculated relative to the expression level on day 28 using GAPDH as a reference gene. Each bar represents mean ± standard error of 3 to 5 chickens. The statistical significance was analyzed using one-way ANOVA followed by Tukey’s Test. **P < 0.01; ***P < 0.001.

In order to study the dynamic expression of four chicken cathelicidins during early development, we collected the bursa, lung, cecum, and cecal tonsil from broiler chickens of 2, 4, 7, 14, and 28 days and then evaluated the expression of cathelicidins using real-time PCR. We observed an obvious differential expression pattern with all four cathelicidins. In the bursa, the expression of cathelicidins peaked 4 days after hatching and was then gradually decreased, with the lowest expression seen on day 28 (Figure 2). A significant decrease (*P* < 0.01) of cathelicidins B1 and fowlicidin-1 were observed in the bursa on day 28 relative to day 4. The expression of fowlicidin-1 was also significantly reduced by nearly 15-fold between day 4 and day 28 (Figure 2). The inverse correlation between the expression of cathelicidins and the maturation of bursa is perhaps not a coincidence, given that T and B cell development is initiated on day 7 in the bursa [23]. It is conceivable that the innate immune mechanism can be dispensable once the adaptive immunity takes control.

**Figure 3:**
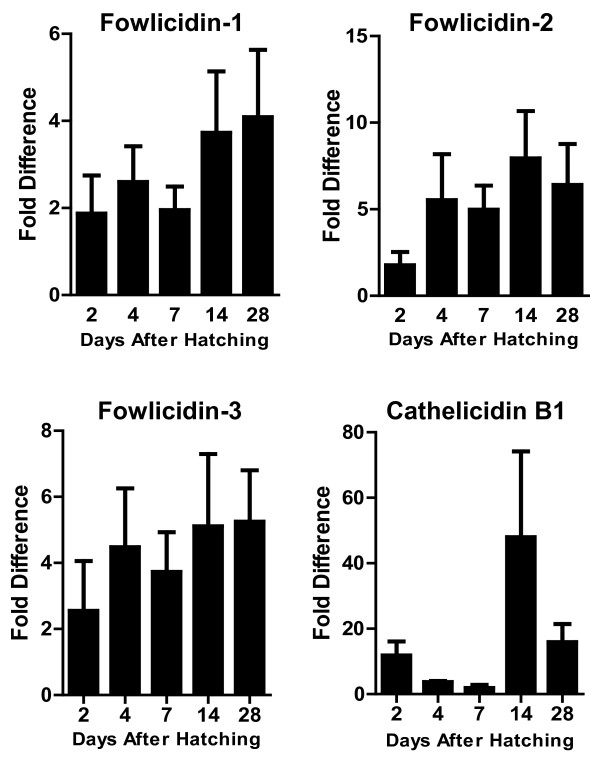
**Developmental Regulation of chicken cathelicidins in the lung.** Lungs were harvested from broiler chickens of indicated age and subjected to RNA isolation and real-time PCR analysis of the expression of four cathelicidins. Fold differences in the cathelicidin expression level among different days after hatching were calculated relative to the expression level on day 2 (for fowlicidins 1 to 3) or day 7 (for cathelicidin B1) using GAPDH as a reference gene. Each bar represents mean ± standard error of 3 to 5 chickens. The statistical significance was analyzed using one-way ANOVA followed by Tukey’s Test.

**Figure 4:**
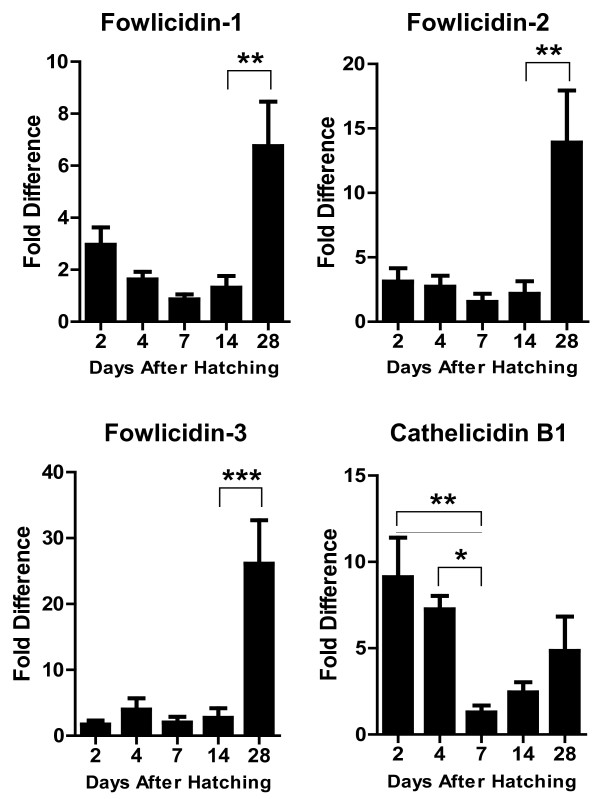
**Developmental Regulation of chicken cathelicidins in the cecum.** Ceca were harvested from broiler chickens of indicated age and subjected to RNA isolation and real-time PCR analysis of the expression of four cathelicidins. Fold differences in the cathelicidin expression level among different days after hatching were calculated relative to the expression level on day 7 using GAPDH as a reference gene. Each bar represents mean ± standard error of 3 to 5 chickens. The statistical significance was analyzed using one-way ANOVA followed by Tukey’s Test. *P < 0.05; **P < 0.01; ***P < 0.001.

**Figure 5:**
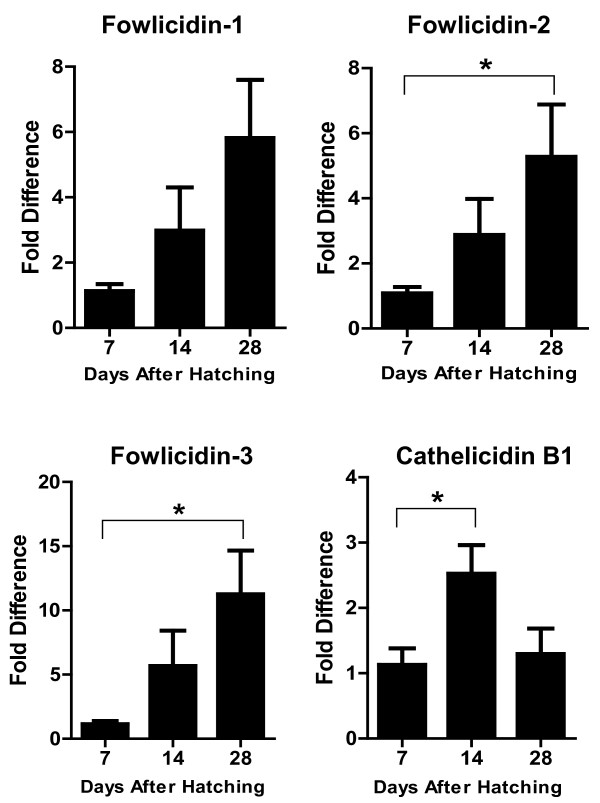
**Developmental Regulation of chicken cathelicidins in the cecal tonsil.** Cecal tonsils were harvested from broiler chickens of indicated age and subjected to RNA isolation and real-time PCR analysis of the expression of four cathelicidins. Fold differences in the cathelicidin expression level among different days after hatching were calculated relative to the expression level on day 7 using GAPDH as a reference gene. Each bar represents mean ± standard error of 3 to 5 chickens. The statistical significance was analyzed using one-way ANOVA followed by Tukey’s Test. *P < 0.05.

Interestingly, a largely opposite developmental expression pattern was seen in the lung, where four cathelicidins showed a tendency to increase the expression level gradually along with the age, with the peak expression occurring on day 14 to 28 (Figure 3). However, none of the differences is statistically significant (*P* > 0.05). In the cecum, a biphasic expression pattern was observed with cathelicidin B1 and fowlicidin-1, where both genes were highly expressed initially on day 2 to 4, but gradually declined to the lowest level on day 7, followed by gradual increase 2 to 4 weeks after birth (Figure 4). On the other hand, a largely constant expression of fowlicidins 2 and 3 were observed till 3 weeks after hatching, and an abrupt increase by 12-to 22-fold was noted on day 28. Cecal tonsils showed an increased expression of fowlicidins 1 to 3 proportional to the age during the first four weeks, whereas the highest expression of cathelicidin B1 occurred on day 14 after hatching, with day 7 and 28 showing reduced expression (Figure 5).

Overall, following a gradual increase in the expression of cathelicidins and many other innate host defense factors in a sterile environment during the embryonic development of chickens [17], we observed a further augmentation of cathelicidin expression during the first 28 days after hatching. The results are perhaps not surprising, given that newly hatched chickens are constantly exposed to various pathogens in ambient environments, albeit with the presence of circulating maternal antibodies, which are acquired through egg yolk, but are insufficient to provide adequate protection against microbial infections [24]. In addition to maternal antibodies, the innate immune mechanisms must be present and develop quickly before the adaptive immunity matures. The availability and enhanced expression of cathelicidins may provide an important protection mechanism against the invading infections during the early stages of life in chickens. Consistently, chicken cathelicidins and other AMPs were shown recently to possess potent antimicrobial activities against a broad range of pathogens including many intestinal bacteria [9,11-14].

However, it remains unclear in our study whether an enhanced expression of chicken cathelicidins during the early development is triggered by developmental signals or a consequence of constant exposure to microflora or environmental pathogens. To dissect it, chickens raised under germ-free conditions have been to be used. Nevertheless, many AMPs including a cathelicidin were detected in human meconium and neonatal fecal extracts, both of which indeed showed direct antibacterial activities [15]. It is tempting to speculate that the presence of AMPs in the neonatal gut not only provides an important host defense mechanism, but also control initial colonization of intestinal flora. The amount and type of each AMP present in the gut may dictate the profiles of microbiota, given several different human AMPs exhibiting overlapping but not identical antimicrobial spectra [25].

In summary, all four cathelicidins are widely expressed in a broad range of chicken tissues, suggestive of their important innate defense role. Moreover, an augmented synthesis of the cathelicidin transcripts during the development coincides with the maturation of the immune system and a need for protection of the host in ambient environments. Our study of tissue and developmental expression of four chicken cathelicidins has shed new lights on the mechanisms of innate host defense and development of the immune system of chickens. Because of an association of single nucleotide polymorphisms (SNPs) in several chicken AMPs with animal resistance to *Salmonella* infection [26,27], it is possible to genetically select chicken lines with enhanced disease resistance. Alternatively, dietary modulation of endogenous AMP expression represents another convenient approach to disease control and prevention in both humans and animals [28,29].

## Competing interests

The authors declare that they have no competing interests.

## Authors’ contributions

MA, LTS, GD, YRB, WJ, and GZ carried out the experiments. MA and GZ participated in the design of the study, performed the statistical analysis, and drafted the manuscript. All authors read and approved the final manuscript.
